# Sugarcane Straw Hemicellulose Extraction by Autohydrolysis for Cosmetic Applications

**DOI:** 10.3390/molecules30061208

**Published:** 2025-03-07

**Authors:** Maria João Pereira, Sílvia S. Pedrosa, Joana R. Costa, Maria João Carvalho, Tânia Neto, Ana L. Oliveira, Manuela Pintado, Ana Raquel Madureira

**Affiliations:** CBQF–Centro de Biotecnologia e Química Fina–Laboratório Associado, Escola Superior de Biotecnologia, Universidade Católica Portuguesa, Rua de Diogo Botelho 1327, 4169-005 Porto, Portugal; mjvpereira@ucp.pt (M.J.P.); jrcosta@ucp.pt (J.R.C.); mjcarvalho@ucp.pt (M.J.C.); taneto@ucp.pt (T.N.); mpintado@ucp.pt (M.P.); rmadureira@ucp.pt (A.R.M.)

**Keywords:** sugarcane straw, hemicellulose valorization, autohydrolysis, XOS production, cosmetic potential

## Abstract

Sugarcane is a popular crop whose cultivation generates a wide range of by-products. The aim was to optimize the hydrothermal extraction of hemicellulose from sugarcane straw using response-surface methods with a two-factor composite design and to assess its functional qualities. Three process parameters were subject to optimization: solid/liquid ratio (1:6–1:18), temperature (143–186 °C), and extraction time (20–60 min). A xylooligosaccharide (XOS)-enriched extract was characterized regarding its chemical composition, molecular weight, and antioxidant and antimicrobial potential. The optimized extraction yield was 24.46 g/100 g of straw with a polymerization degree of 17.40. Both hemicellulose and XOS demonstrated notable antioxidant properties, with antioxidant effects of 73% and 85%, respectively. Regarding skin enzyme activity, hemicellulose inhibited elastase by more than 50%, while XOS showed no significant effect. However, both extracts exhibited collagenase (MMP1) inhibition comparable to the positive control. In terms of production feasibility, the estimated costs were 130.5 EUR/kg for hemicellulose and 272.5 EUR/kg for XOS. Overall, the optimized XOS-enriched sugarcane straw extract demonstrated promising anti-aging, antioxidant, and preservative properties, highlighting its potential for cosmetic applications.

## 1. Introduction

The circular bioeconomy has recently gained increased attention in several countries because of the new legislation concerning the reuse, valorization, and long-term viability of biomass as a raw resource [1,2].

A large portion of the lignocellulosic residues produced by agriculture is used as feedstock in fractionation and hydrolysis operations to create fermentable sugars. In countries like Brazil, China, India, Thailand, and Australia, flat ground is predominantly used for the cultivation of sugarcane as an energy crop. Straw, also known as cane trash, is one of the primary by-products of sugarcane processing and produces 140 kg of every tonne of sugarcane processed. Straw is made up of dry, green leaves and tips [2,3]. Brazil alone is expected to produce 652.9 metric tonnes of sugarcane in the 2023/2024 crop, and at least 20 million tonnes of sugarcane straw (SCS) biomass be harvested for biorefinery [4,5]. The majority of SCS is made up of cellulose (31–45%), lignin (12–31%), and hemicelluloses (20–30%). Extractives (4–16%) and ashes (2–8%) are other less-represented elements [3,4,6].

The four types of hemicellulose structures are xyloglucans, mixed-linkage glucans, mannans, and xyloglucans. The backbone of xylose residues, which form the linear hemicellulose-xylans, commonly substituted with other oligosaccharides or small polysaccharides such as xylose, arabinose, glucose, and glucuronic acid. Additionally, minor groups can be attached to the side chains or backbone of xylans, such as phenolics, acetyl groups, and ferulic and coumaric acids. The primary hemicellulose in hardwood biomass is called xylan, and it is connected to secondary cell wall composition and structure—which are linked to biomass resistance [7,8,9].

The production of xylooligosaccharides (XOSs), which are xylose oligomers mostly resulting from hemicellulose found in plant biomass (like sugarcane straws), has recently received significant interest [10,11]. XOSs with a degree of polymerization (DP) from 2 to 20 units are reported to have applicability as low-calorie sweeteners for sugar and fat substitutes, the ability to retain moisture, prebiotic qualities, a pH from 2.5 to 8, and temperature stability up to 100 °C [4,12]. These properties can be targeted and are useful in health supplements.

The biomass fractionation can occur by different types of pre-treatment procedures: (1) physical methods, such as milling and grinding; (2) chemical, like alkali, acid, organosolvant, and ionic liquid extraction; (3) physicochemical, such as ammonia fiber expansion and steam explosion; or (4) biological, by using enzymes or microorganisms [1,4,7,9,13]. Autohydrolysis, also known as hydrothermal processing, is a physicochemical process that is regarded as an environmentally friendly technique, motivated by the fact that it only needs water as a solvent in the process. The selective extraction of hemicellulose and the lack of equipment corrosion are the main advantages of this method, which makes autohydrolysis of hemicellulose from lignocellulosic-rich biomass an efficient fractionation process to produce xylooligosaccharides [6,7,14]. During autohydrolysis, the hydronium ions, produced by water and acetic acid ionization, act as a catalyst for the breakdown of polysaccharides. Due to its ability to solubilize hemicellulose without the requirement of an extra catalyst, the autohydrolysis process is a particularly intriguing fractionation method. The acetyl groups contained in xylans in the form of esters are mostly attacked by the protons produced by the autoionization of water [14].

Further separation procedures can be used to increase the purity levels of XOS. Some of these methods include centrifugation, filter filtering, molecular weight filtration (MWCO), decolorization, and spray drying. The degree to which compounds are soluble relies on their intermolecular bonding, molecular weight, and overall solubility. As stated by the anticipated final product’s use and nature, recognized oligosaccharide purification processes include flocculation. centrifugation, ion exchange, nanofiltration, ultrafiltration, charcoal adsorption, reverse osmosis, and vacuum evaporation [13].

The XOS extraction from industrial and agricultural wastes, if economically viable, could represent an important commercial product/ingredient. Despite their importance and benefits for human health, XOSs are relatively expensive, with market prices ranging from USD 22 to USD 50/kg depending on the purity level [4,13]. Currently, XOSs are widely used in several industries, such as the pharmaceutical, food and feed, and health industries. Additionally, motivated by their antioxidant and antimicrobial potential, they may have an interesting application as cosmetic ingredients [1,15].

This work aims to valorize the hemicellulose in sugarcane straw by autohydrolysis treatment. A central composite experimental design was used to evaluate how the S/L ratio and temperature affect the composition of the resulting autohydrolysis liquor. The yield and degradation products generated are quantified and used to determine the best operating conditions to produce an XOS-enriched product. Additionally, the effect of extraction time was evaluated using the same parameters. For purification purposes, an ultrafiltration purification step was tested. With the optimized conditions, the XOS-enriched products were characterized regarding their cosmetic potential application and their process economic viability.

## 2. Results and Discussion

### 2.1. Optimal Autohydrolysis Conditions of Hemicellulose from Sugarcane Straw

#### 2.1.1. Sugarcane Biomass Characterization

The chemical composition of the sugarcane straws was 36.29% of cellulose, 24.83% of hemicellulose (21.64% xylan and 3.19% arabinan), 17.91% of lignin, and 1.41% of ashes. This composition varies depending on the location, plant growth conditions, climate, soil type, and type of tissue analyzed [16]. The results agree with data reported Costa et al. [17]. Sugarcane contains a high proportion of xylan, making it a suitable feedstock for XOS biosynthesis. Another benefit is that sugarcane straw has no effective use besides being left in the fields and available in several countries [18].

#### 2.1.2. Solid/Liquid Ratio and Temperature

**Figure 1:**
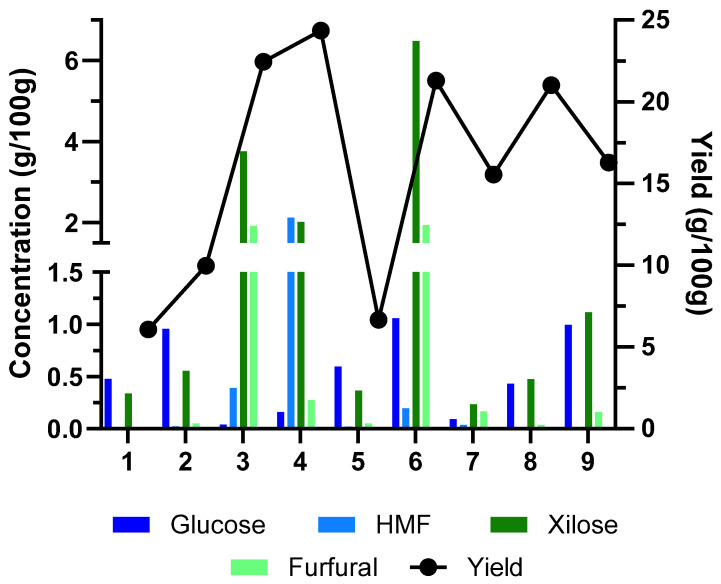
Results of monosaccharide and degradation products in hemicellulose after autohydrolysis and yield of hemicellulose extraction from sugarcane straw using S/L ratio and temperature as variables.

The effect of the solid/liquid ratio and temperature conditions of autohydrolysis were studied, with a focus on the yield, presence of monosaccharides, and degradation products generated. The results are presented in Figure 1 and Table 1.

The yield of the liquid fraction varied significantly across the experimental runs (S/L ratio of 1/8), ranging from 6.07% to 24.34%. The highest yields were observed at 180 °C (Runs 3 and 4), while lower yields were obtained at lower temperatures (150 °C—Runs 1 and 2) and at Run 5 (143 °C). Increasing the temperature from 150 °C to 180 °C resulted in a substantial increase in the liquid fraction yield. This suggests that higher temperatures promote the breakdown of the sugarcane straw matrix and the release of soluble compounds. Xylose was the predominant sugar in the liquid fraction across all runs, indicating the effective hydrolysis of hemicellulose. Similar to the total yield, xylose concentration generally increased with temperature, peaking at 186 °C (Run 6) before slightly decreasing at 165 °C (Run 7).

**Figure 2:**
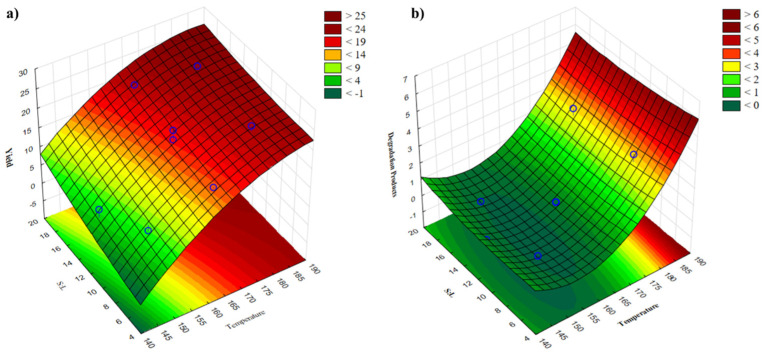
Response-surface plot for yield (**a**) and degradation products (**b**) according to the experimental design.

After 11 experiments with extraction yields and degradation products as the dependent variables, a central composite design was employed. The regression coefficients and the results of the analyses of variance (ANOVA) for the second-order polynomial models are compiled in Table 2. Effects were classified as significant if their *p*-values were less than 0.05 at the 95% confidence level. According to the polynomial model that was created for each parameter under consideration, the surface response is displayed in Figure 2. The variables that were displayed had the greatest and most notable impacts on the response.

The behavior observed in Table 1 and Figure 2 shows that temperature has a significant impact on both dependent variables. The solid/liquid ratio does not show a significant impact on both dependent variables but rather works as a co-adjuvant. Higher temperature results in a higher yield. However, higher temperatures cause an increase in sugar degradation products such as 5-hydroxymethylfurfural (HMF) and furfural, which is not desirable. High temperatures and/or prolonged heating cause a loss of xylose or arabinose, most likely converted into furfural or reacted with peptide amino groups to produce Maillard browning products with a dark color and a burnt-sugar odor. On the other hand, when hemicellulose is autohydrolyzed under certain operating circumstances, xylooligosaccharides (XOSs) are the predominant product, accounting for approximately 80% of the total sugars solubilized in the autohydrolysis liquid phase formed [19].

Aiming to extract a mixture rich in XOSs, the molecular weight of hemicellulose liquor obtained in the different experimental conditions was characterized (see Table 3). As the autohydrolysis temperature increased from 143 °C (Runs 5) to 186 °C (Run 6), and the average molecular weight of the hemicellulose extracted decreased from 126.29 kDa to 20.96 kDa. This indicates that higher temperatures promote the breakdown of hemicellulose into smaller fragments. The operation conditions that provide a higher molecular weight dispersity are temperatures between 143 and 165 °C. The S/L ratio does not show an impact on the molecular weight dispersity.

Considering the extraction yield of each experimental condition tested, the contaminant content (monosaccharides and degradation products), and the molecular weight dispersity, the best overall conditions were found to be a 1:18 solid/liquid ratio and 165 °C.

#### 2.1.3. Hemicellulose Extraction Time

**Figure 3:**
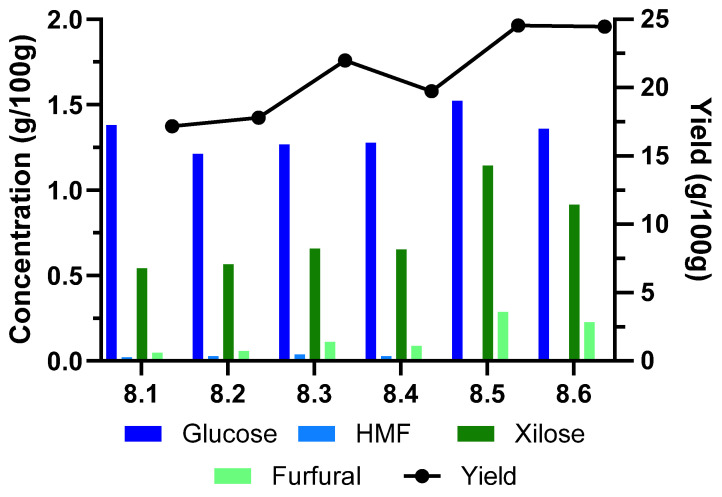
Monosaccharide and degradation products in hemicellulose after autohydrolysis and yield of hemicellulose extraction from sugarcane straw using time of extraction as a variable.

Following a similar strategy, optimization of the extraction time was performed with extraction yield, molecular weight, the presence of monosaccharides, and degradation product as key outcomes. The experimental setup is detailed in Figure 3 and Table 4, as well as the hemicellulose yields and the characterization of monosaccharide and degradation products present in the hemicellulose liquor.

Regarding the obtention of a higher yield of hemicellulose extraction, the optimum time of extraction found was 60 min. However, the longer reaction time produces a higher concentration of degradation products in the post-hydrolysis process. These results agree with Egüés et al. (2012) [7], who obtained greater degradation of the compounds (the acetic acid content reached a maximum value of 0.84 g/L at 200 °C and 30 min, respectively).

The best operation conditions for a higher extraction yield reducing the amount of degradation products were applying a temperature of 165 °C, an S/L ratio of 1/18, and an extraction time of 45 min.

### 2.2. Hemicellulose Liquor and Purified XOS Extract from Sugarcane Straw Physicochemical Properties

After autohydrolysis, the hemicellulose liquor from the sugarcane straw was filtered (5 mm screen) to eliminate all insoluble particles before being lyophilized. The results of the sugar content of the straw hemicellulose fractions as determined by the National Renewable Energy Laboratory (NREL) method and the molecular weight as determined by HPLC-RI are presented in Table 5 and Table 6. A purification step of ultrafiltration (3 kDa mesh) was performed to obtain an XOS-enriched extract. Both extracts were characterized and analyzed as potential cosmetic ingredients.

After 45 min of autohydrolysis at 165 °C, xylose is the most prevalent sugar in the hemicellulose extract, indicating that xylan is the predominant polysaccharide. For hemicellulose extract, minor quantities of arabinose and glucose are available.

Similar results are obtained for the XOS-enriched extract after the ultrafiltration step. Similar results were obtained by applying 160 °C and 30–60 min of extraction with wheat straws by autohydrolysis [19]. The hemicellulose fraction was composed mainly of xylose (29.4%), arabinose (1.9%), and acetyl groups (2.5%). The hemicellulose extract also presents acetic acid, which is a product that is derived from the hydrolysis of hemicelluloses and acts as a carbohydrate catalyst [20].

The biological properties of XOS are influenced by their molecular weight distribution, as well as the type and degree of substitution [21]. The XOS-enriched extract showed an average molecular weight (MW) and number average molecular weight (Mn) of 14.11 and 2.58 kDa, respectively. The polymerization degree (DP) obtained in the XOS-enriched extract indicates that ultrafiltration was a successful purification step, since DP values below 20 classify oligosaccharides as XOSs [12].

**Figure 4:**
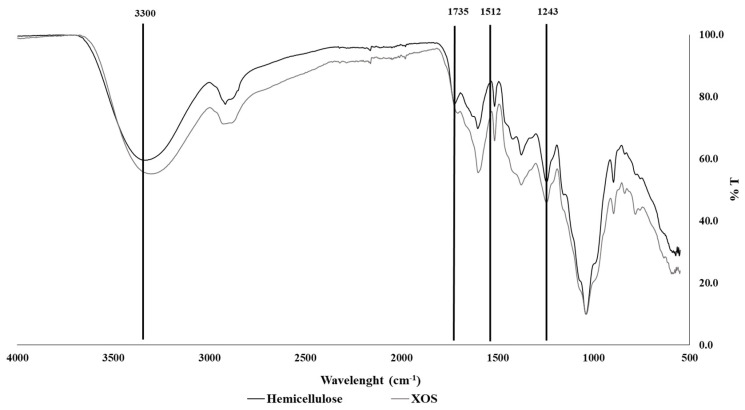
FTIR spectrum of straw hemicellulose and XOS extract.

The functional groups of the hemicellulose and XOS extracts prepared under the optimal conditions (165 °C for 45 min) identified by FTIR are shown in Figure 4.

Both extracts analyzed by infrared spectra displayed the normal signal pattern for hemicellulose fractions. The water’s hydroxyl (-OH) groups are stretched as shown by the band at 3300 cm^−1^ [22,23]. The acetyl group (C=O) of hemicellulose structure and the ester bond of ferulic acid carboxyl stretching group are both present in the band at 1725 cm^−1^ [24]. The two absorbances in 1512 and 1243 cm^−1^ are characteristic bands of lignin and their aryl–alkyl ethers (O-CH_3_), indicating that hemicellulose was contaminated with amounts of bound lignin [22]. Montané et al. (2006) reported that XOSs produced by autohydrolysis contain lignin-derived phenolic impurities [25].

#### Organic Acids and Phenolic Compounds Profile in XOS-Enriched Extracts

Phenolic compounds and organic acids of XOS-enriched extract were identified and quantified by LC-MS. This method allowed the identification of many classes of compounds. In this case, the main components are organic acids and, in terms of polyphenols, hydroxybenzoic and hydroxycinnamic acids (Table 7).

The major class of compounds identified in XOS-enriched extract was organic acids, which was expected since hemicellulose has been studied and used to produce several organic acids [26]. Additionally, during the production of XOS, severe circumstances caused the hemicellulosic sugars to breakdown, which led to the creation of inhibitory substances such as furfural and organic acids [27].

Ferulic acid and *p*-coumaric are reported as the main hydroxycinnamic acids found in sugarcane [28], which was confirmed in both extracts from sugarcane straws. Due to their antioxidant potential and health advantages, hydroxycinnamic acids are increasing in popularity in medicinal, industrial, and food applications [28].

A concentration effect, or an increase in the amount of all phenolic and organic acid components previously found in the hemicellulose extract, was the result of the purifying procedure. This result was anticipated since contaminants larger than 3 kDa, such as lignin, cellulose particles, and others, were eliminated [29,30].

Phenolic compounds, such as caffeic acid, *p*-coumaric acid, and ellagic acid, contribute to the antioxidant properties of XOS, which can help to protect the skin from oxidative stress and aging [31]. These compounds also exhibit anti-inflammatory effects, potentially benefiting skin health and reducing signs of aging [32]. Organic acids, including malic acid and citric acid, can enhance the stability and bioavailability of XOSs, improving their efficacy in cosmetic formulations [33]. The combination of phenolic compounds and organic acids in XOSs may synergistically boost their antioxidant capacity, making them valuable ingredients in skincare products for their potential to improve skin elasticity, reduce wrinkles, and promote overall skin health [32,34].

### 2.3. Antioxidant Activity

**Figure 5:**
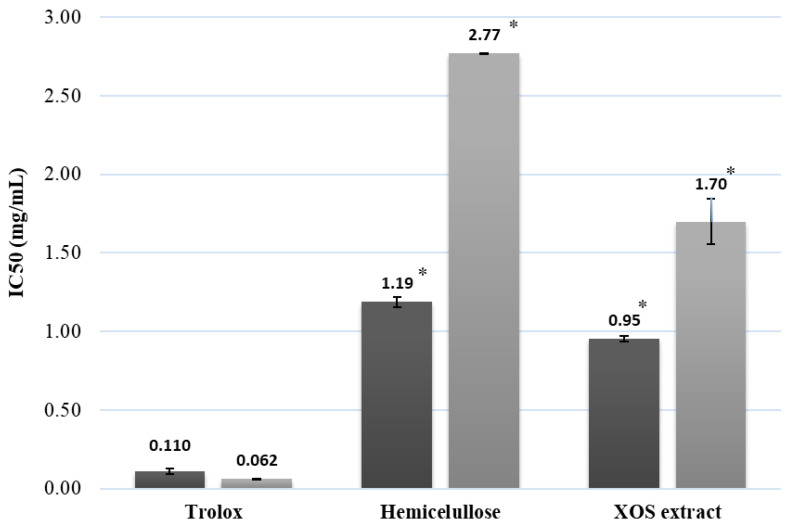
Antioxidant activity of hemicellulose and XOS extracted from sugarcane straw. Asterisk on a graph represents statistical differences between groups.

Antioxidant compounds often work differently depending on the conditions of the interaction, including the kind of molecule and the oxidizing agents they interact with. A complex system’s antioxidant capacity cannot be precisely measured by a single approach; thus, the antioxidant capacity must be approximated using a variety of techniques [35]. In this context, the ability of a sample to transfer electrons or hydrogen atoms is utilized to measure antiradical activity in the commonly used DPPH and ABTS assays. The scavenging capacity of hemicellulose and XOS-enriched extracts from the autohydrolysis of sugarcane straws is shown in Figure 5.

Antioxidant activity was assessed using both ABTS and DPPH assays to account for the different reaction mechanisms involved in free radical scavenging. In general, higher IC50 values were observed in the DPPH assay compared to the ABTS assay for all tested samples (Figure 5). This may be attributed to differences in the reaction kinetics or the specific types of radicals targeted by each assay. The ABTS assay measures the antioxidant’s ability to transfer an electron, while the DPPH assay measures its ability to donate a hydrogen atom. The discrepancy between the assays suggests that the antioxidants present in the hemicellulose and XOS extracts may be more effective at electron transfer than at hydrogen atom donation. This observation aligns with the findings of Oliveira et al. (2002) [36], who attributed this difference to the presence of compounds in sugarcane straw extracts that are more effective at transferring electrons (as measured by ABTS) than donating hydrogen atoms (as measured by DPPH). This suggests that the antioxidant mechanism of sugarcane straw hemicellulose and XOS may primarily involve electron transfer.

### 2.4. Antimicrobial Activity in Skin Microorganisms

Regarding the potential application of XOS-enriched extract in cosmetic formulations, the microorganisms selected for this study were generally found on the skin microbiota. Table 8 displays the results of the determination of XOS’s minimum inhibitory concentration (MIC) and minimum bactericidal concentration (MBC).

The XOS-enriched extract showed an antimicrobial effect on 6 of the 11 microorganisms tested and in the case of *A. johnsonii*, a bactericidal effect was even achieved at 3% concentration. XOSs’ antibacterial activity against a range of microbial species has been documented and regarded as one of the most important features directly related to their biological uses [37].

The phenolic compounds, which have been shown to have several physiological advantages such as antibacterial, anti-inflammatory, and antioxidant properties, may be the source of this antimicrobial activity [38].

The antimicrobial activity against *E. coli* and *P. aeruginosa* indicates that the XOS-enriched extract can be used as a preservative in skin care products to increase their stability and shelf life.

Even though *C. jeikeium* and *A. johnsonii* are commonly residents of the skin, these can lead to nosocomial infection [39,40]. Thus, antimicrobial activity against these agents can contribute to preventing these types of infections. Activity against *T. cutaneum* can also be beneficial since this yeast is responsible for provoking hair infection [41], whereas activity against *S. epidermidis* can be somewhat concerning since this bacteria has an important role in maintaining skin microbiota homeostasis [42].

### 2.5. Cosmetic Potential

#### 2.5.1. Cytocompatibility

The viability of dermal fibroblasts (HDFas) and keratinocytes (HaCaTs) exposed to different concentrations of hemicellulose and XOS-enriched extracts was investigated. The hemicellulose extract showed a cytotoxic effect at 0.125 and 0.1% for HDFa and HaCaT cells, respectively. Concerning the XOS-enriched extract, a cytotoxic effect occurs at 0.1% in HaCaT cells and 0.08% in HDFa cells.

#### 2.5.2. Skin Enzymes Inhibition

In the skin ageing process, an alteration in the balance of skin enzyme production and degradation usually occurs. This phenomenon has an impact on the regulation of skin strength and elasticity. Collagenase is an enzyme that degrades collagen and is responsible for skin elasticity and strength loss. Elastin is another crucial protein that contributes to the skin matrix’s elasticity. And, while tyrosinase is an important enzyme in melanin formation, it can induce hyperpigmentation owing to melanin build-up in particular areas of the skin [43]. The inhibitory effect of hemicellulose liquor and XOS extracts on skin enzymes—tyrosinase, neutrophil elastase, and MMP1 (collagenase)—was evaluated by colorimetric or fluorometric enzyme inhibition screening tests from Abcam^®^ (Cambridge, UK).

**Figure 6:**
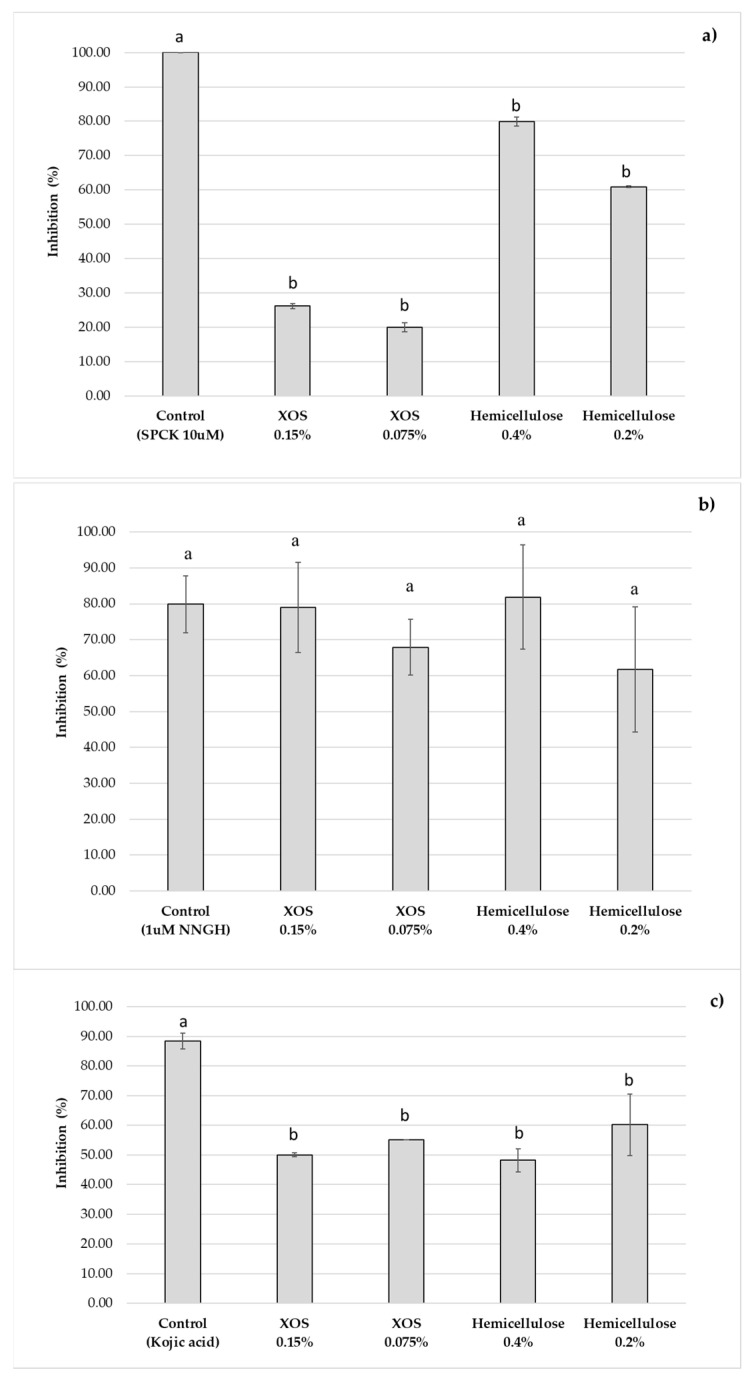
The inhibitory effect of hemicellulose and XOS extracts on skin enzymes: (**a**) neutrophil elastase, (**b**) collagenase (MMP1), and (**c**) tyrosinase. Letters on a graph represent statistical differences between groups. Different letters indicate significant differences, while the same letter means no significant difference.

Both extracts obtained generally demonstrated the effective inhibition of skin enzymes, suggesting a possible application in anti-aging formulations. The hemicellulose extract had a higher inhibitory rate (>50%) than the XOS-enriched extract in the case of the elastase enzyme. Even though the extracts were evaluated at different concentrations, the inhibitory reaction on the enzyme’s collagenase and tyrosinase was similar, being more concentrated in the hemicellulose extract. To further emphasize the point, the collagenase inhibition percentage obtained was remarkable and comparable to the positive control (Figure 6).

#### 2.5.3. Compatibility with Cosmetic Ingredients

To evaluate the potential use of hemicellulose and XOS-enriched extracts produced from sugarcane straws as cosmetic ingredients, the compatibility of these extracts with several common ingredients currently used in cosmetic formulations was evaluated by FTIR (see Figure S1 in the Supplementary Materials).

Different types of ingredients were tested including a thickener agent (Shea butter, Acofarma, Madrid, Spain), emulsifier (Miglyol 812, Acofarma, Madrid, Spain), emollients (Petrolatum (LabChem, Loures, Portugal), Mineral Oil (Sigma-Aldrich, St. Louis, MO, USA), and Cetiol V Acofarma, Madrid, Spain), and preservative (phenoxyethanol, Sigma-Aldrich, St. Louis, MO, USA). Generally, the XOS-enriched extract was compatible with all the cosmetic ingredients tested, except for Mineral Oil.

### 2.6. Economic Viability

Evaluating the possibility of the large-scale production of hemicellulose and XOS-enriched extracts requires a thorough investigation of its economic viability. Table 9 and Table 10 presents the overall estimated production costs of both extracts studied, which were EUR 130.57/kg and EUR 272.57/kg for hemicellulose and XOS-enriched extract, respectively. The cost difference between the two extracts was justified by the yield achieved after the ultrafiltration step. The cost of the membranes, which could increase the final cost, was not considered since there is a possibility of reuse on an industrial scale.

Lower yields are more common in lab-scale operations due to either process-related mass losses or the reduced yield of specific lab-scale equipment. While process mass balances were achieved using lab-scale production, chemical and energy costs were based on bulk quantity pricing to provide a more realistic simulation. Further research with pilot-scale data is required to reach at a more realistic cost.

## 3. Materials and Methods

### 3.1. Raw Material and Materials

The sugarcane straw (SCS), harvested in October 2018, was supplied by Raízen (São Paulo, Brazil), situated at Brotas, Brazil. SCS was oven-dried (40 °C for 24 h) and milled (Retsch, Germany), and fractionated using 150 and 900 μm sieves. Biomass with particle sizes between 150 and 900 μm was selected for further study.

### 3.2. Experimental Procedure

**Figure 7:**
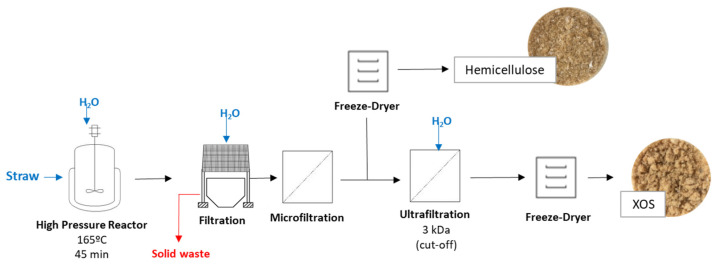
Flow chart summarizing the process to obtain hemicellulose and XOS fractions from sugarcane straw.

Figure 7 illustrates the flow of the experiments conducted in this work. To obtain hemicellulose and XOS purified fractions from sugarcane straw, autohydrolysis followed by membrane filtration was proposed.

The process of obtention of hemicellulose fraction and further purification for recovery of the XOS fraction was performed in two major steps:

In the **autohydrolysis step**, studies were performed aiming to determine the best operating conditions to extract hemicellulose from the sugarcane straw. Two independent variables (solid (straw)/liquid (water) (S/L) ratio and temperature) and a dependent variable (extraction time) were assessed.

In the **purification step** by ultrafiltration, studies were conducted aiming at the separation of the XOS fraction of the hemicellulose fraction, through ultrafiltration using a cut-off of 3 kDa.

#### 3.2.1. Autohydrolysis Process

The optimization of sugarcane straw autohydrolysis was carried out in a Parr reactor with a 4551-reactor controller (4551-T-SS-HD-230-VS.50-1000-4848, Parr Instrument Company, Moline, IL, USA) in a 360 mL volume. In accordance with the experimental conditions, the biomass was combined with deionized water (Table 11). The manufacturer’s instructions were followed when setting up the stirred Parr reactor. The temperature was set according to the experimental conditions and agitation was set at 100 rpm (Position 1) for all runs.

##### Experimental Design

Using the hemicellulose extraction yield as a response, the optimal extraction parameters were adjusted in accordance with a central composite design (CCD). Two independent variables were assessed, solid (straw)/liquid (water) (S/L) ratio and temperature, and their ranges were 1:6–1:18 and 143–186 °C, respectively. The complete design consisted of 11 experimental trials (see Table 1). The following equation (Equation (1)) was used:

(1) $$y=\beta_{0}+\beta_{1}x_{1}+\beta_{2}x_{2}+\beta_{11}x_{1}^{2}+\beta_{22}x_{2}^{2}+\beta_{12}x_{1}x_{2}$$

where $\beta_{n}$ represents the constant regression coefficients, *y* is the hemicellulose extraction yield, and $x_{1}$ and $x_{2}$ are the independent variables—S:L ratio and temperature, respectively.

**Table 11:** Description of the experimental conditions used for hemicellulose extraction optimization according to the central composite design.

Run	ϰ_1_—Temperature (°C)	ϰ_2_—S:L Ratio	Initial Weight (g)	Time (min)
1	150 (−1.0)	1:8 (−1.0)	45	15
2	150 (−1.0)	1:16 (+1.0)	22	
3	180 (+1.0)	1:8 (−1.0)	45	
4	180 (+1.0)	1:16 (+1.0)	22	
5	143 (+1.41)	1:12 (0)	30	
6	186 (+1.41)	1:12 (0)	30	
7	165 (0)	1:6 (−1.41)	60	
8	165 (0)	1:18 (+1.41)	20	
9	165 (0)	1:12 (0)	30	
10	165 (0)	1:12 (0)	30	
11	165 (0)	1:12 (0)	30	

The impact of extraction time was evaluated after determining the best conditions for the S/L ratio and temperature experimental conditions. Application of the optimal conditions previously studied (Run 8—S/L ratio of 1:18 and a temperature of 165 °C) was used for the further optimization of extraction time. The experimental conditions tested are described in Table 12, and the results were analyzed based on yield and average molecular weight.

#### 3.2.2. Purification of XOS Fractions

To fractionate the hemicellulose in xylan and XOS fractions, the hemicellulose liquor was processed by ultrafiltration with a cut-off of 3 kDa. A volume of 400 mL of hemicellulose was filtrated through a 3 kDa mesh and the retained fraction was washed with 3-times the initial volume (1200 mL). The XOS extract was characterized by LC-ESI-QqTOF-HRMS for organic acid and phenolic compound composition.

### 3.3. Analytical Characterization

#### 3.3.1. Quantification of Lignin, Cellulose, and Hemicellulose by Nrel Protocol

Characterization of sugarcane straw was established using the National Renewable Energy Laboratory (NREL) standard [44]. Using concentrated sulfuric acid (72% *w*/*w*) at 30 °C for an hour in a water bath is the first stage in the acid hydrolysis process. To halt the reaction, deionized water was added to a specific mass of the samples after they had been carefully moved to a Schott flask. The samples were then autoclaved for one hour at 121 °C. The suspension was filtered with a G3 crucible previously calcined at 550 °C. The liquid fraction was stored and sugar and soluble lignin quantified. The precipitate was thoroughly washed to remove the acid solution. The crucibles containing the klaxon lignin (insoluble lignin) were placed in an oven at 105 °C overnight. After, they were weighed, and the determination of ash content was performed after calcination at 550 °C. The soluble lignin was quantified by UV–Vis spectrophotometry (UV-1900 Shimadzu, Kyoto, Japan) at 205 nm.

#### 3.3.2. Modified NREL Protocol—Post Hydrolysis

A post-hydrolysis procedure using sulfuric acid (4% *w*/*w*) at 121 °C for 30 min was conducted to determine the amount of oligosaccharides in the liquid phase, based on the modified National Renewable Energy Laboratory (NREL) protocol described in the literature [21,45].

#### 3.3.3. Analysis of Degradation Products by HPLC

Using an Aminex 87-H column and a refractive index detector, the HPLC Agilent 1260 Infinity II LC System [46] (Agilent Technologies, Santa Clara, CA, USA) was used to directly analyze the liquors to measure the concentration of acetic acid, furfural, hydroxymethylfurfural, and monosaccharides that were byproducts of the extraction process.

#### 3.3.4. Size Exclusion Chromatography (SEC)

Using a Refractive Index Detector and an Agilent 1260 Infinity II LC System (Agilent Technologies, CA, USA), the molecular weight of the hemicellulose produced by autohydrolysis was measured. A PL aquagel-OH Mixed-M, 4.6 × 250 mm, 8 µm column and a PL aquagel-OH, 7.5 × 300 mm, 20.5 µm column were operated at 30 °C with ultrapure water as the eluent at a flow rate of 0.5 mL/min. The technique was calibrated using the pullulan P-82 standards (from Showa Denko K. K., Tokyo, Japan), ranging from P-5 (5.9 kDa) to P-800 (708 kDa).

#### 3.3.5. Fourier-Transform Infrared Spectroscopy (FTIR)

Direct transmittance measurements were made using the KBr pellet method on a Perkin-Elmer 16 PC spectrometer (Perkin-Elmer, Boston, MA, USA). Each spectrum was recorded in 20 scans across a frequency range of 600 to 4000 cm^−1^. Averages of the signals were acquired at a resolution of 4 cm^−1^. The samples were ground using spectroscopic grade potassium bromide (KBr), which had been oven-dried prior, to avoid interference caused by the presence of water. Additionally, background spectra were acquired prior to every sample.

#### 3.3.6. Phenolic Compounds and Organic Acids Analysis by LC-ESI-QqTOF-HRMS

Thermo Scientific’s LC-ESI-UHR-QqTOF-MS with the UHPLC Ultimate 3000 was used to analyze the XOS-enriched products from sugarcane straws. Qq-time-of-flight (UHR-QqTOF) with 50,000 full-sensitivity resolutions (FSR) was conducted using a mass spectrometer (Im-pact II, Bruker Daltonics, Bremen, Germany). A 100 mm × 2.1 mm, 2.2 m Acclaim RSLC 120 C18 column was used.

Solvent A was acetonitrile with 0.1% formic acid and Solvent B was 0.1% aqueous formic acid, acting as the mobile phases used in a gradient of 0.25 mL min^−1^, 0 min, 0% B; 10 min, 21.0% B; 14 min, 27% B; 18.30 min, 58%; 20.0 min, 100%; 24.0 min, 100%; 24.10 min, 0%; and 26.0 min, 0%.

### 3.4. Antioxidant Potential Evaluation

#### 3.4.1. ABTS Radical Cation Decolorization Assay

The 2,2′-azino-bis (3-ethylbenzothiazoline-6-sulphonic acid) diammonium salt radical cation (ABTS) decolorization assay was performed as described by Gonçalves et al. (2009) [47]. An ABTS solution of 7 mmol/L of ABTS (Sigma, St. Louis, USA) and a 2.45 mM solution of potassium persulfate (K_2_S_2_O_8_) solution (Merck, Darmstadt, Germany) were prepared at a 1:1 (*v*/*v*) proportion. An initial OD of 0.700 ± 0.020 at 734 nm was obtained by storing the solution in the dark and then diluting it with dH_2_O. In total, 15 μL of each sample was incubated with 200 μL of ABTS for five minutes at 30 °C after the samples were generated at five different concentrations, beginning with a 5 mg/mL concentration. The OD was measured at 734 nm following the incubation time using a Synergy H1 microplate reader (Biotek, Winooski, VT, USA). Trolox (Sigma-Aldrich, St. Louis, MO, USA) was used to create the calibration curve, which ranged from 0.075 to 0.008 mg/mL. The half maximum inhibitory concentration (IC50) (mg/g dry weight) was used to display the results.

#### 3.4.2. DPPH Radical Cation Decolorization Assay

A 2,2-diphenyl-1-picrylhydrazyl (DPPH) assay was performed as Gonçalves et al. (2009) [47]. A 600 μM solution of the 2,2-diphenyl-1-picrylhydrazyl (DPPH) free radicals was prepared by dissolving DPPH (Sigma-Aldrich, St. Louis, MO, USA) in methanol. The solution was then diluted with methanol to achieve an optical density (OD) of 0.600 ± 0.100 at 515 nm. Samples (25 µL), starting at a concentration of 5 mg/mL, were diluted successively and incubated with 175 μL of the DPPH solution for 30 min. The OD at 515 nm was measured using a Synergy H1 microplate reader (Biotek, Winooski, VT, USA). A calibration curve was generated using Trolox (Sigma-Aldrich, St. Louis, MO, USA) at concentrations ranging from 0.075 to 0.008 mg/mL. The results were expressed as IC50 (mg/g dry weight).

### 3.5. Antimicrobial Activity Determination in Skin Microorganisms

#### 3.5.1. Microorganisms

The antimicrobial activity of XOS was studied against common bacteria and yeasts of skin, namely *Staphylococcus aureus* (DSM 799), *Staphylococcus epidermidis* (LMG 10474), *Pseudomonas aeruginosa* (DSM 1128), *Escherichia coli* (DSM 1576), *Corynebacterium jeikeium* (DSM 7171), *Acinetobacter johnsonii* (DSM 6963), *Cutibacterium acnes* (DSM 1897), *Candida albicans* (DSM 1386), *Rhodotorula mucilaginosa* (DSM 70403), *Cutaneotrichosporon cutaneum* (DSM 27285), and *Malassezia furfur* (DSM 6170).

#### 3.5.2. Minimal Inhibitory and Bactericidal Concentrations Evaluation

The minimum inhibitory concentration (MIC) and the minimum bactericidal concentration (MBC) of XOS were determined employing a broth microdilution assay, according to the standards for aerobic bacteria [48], anaerobic bacteria [49], and yeasts [50].

A 3% (*w*/*v*) stock solution was prepared in the appropriate medium and filtered through a 0.22 μm filter (Frilabo, Maia, Portugal). Solutions at concentrations of 2%, 1%, and 0.5% (*w*/*v*) were then prepared and tested. The microorganisms were inoculated at 2% (*v*/*v*) from a standardized inoculum based on the 0.5 McFarland standard (approximately 10^8^ CFU/mL), and the final inoculum concentration was adjusted to 10^5^ CFU/mL in the test solutions. The assay was conducted in a 96-well microplate (Sarstedt, Nümbrecht, Germany) for 24 h at 37 °C for aerobic bacteria. For anaerobic bacteria, incubation was carried out for 48 h at 37 °C with the microplate sealed under a layer of parafilm (Lab Chem, Zelienople, PA, USA) to maintain anaerobic conditions. Yeasts were incubated for 48 h at 30 °C. The optical density (OD) was measured hourly at 660 nm using a Synergy H1 microplate reader (BioTek, Winooski, VT, USA). All measurements were performed in triplicate.

Afterwards, the concentrations for which no growth was detected were plated in the appropriate medium to validate the MBC.

### 3.6. Cosmetic Potential Assessment

#### 3.6.1. Cytocompatibility Evaluation Protocol

HaCaTs (CLS, Lot No. 300493-4619) and Normal Human Dermal Fibroblasts (nHdFs, Cat. CC2511, Lot No. 0000577924) from adult skin (Lonza Bioscience, Basel, Switzerland) were cultured in Gibco Dulbecco’s Modified Eagle Medium (DMEM) supplemented with FBS (10082-147, Gibco), and penicillin (100 U/mL)–streptomycin (100 μg/mL) (15240–062, Gibco, Thermo Scientific, Waltham, MA, USA). Cells were maintained at 37 °C in a 5% CO_2_ humidified atmosphere.

After being seeded at a density of 1 × 10^3^ cells/well in 96-well plates, the cells were allowed to adhere for the whole night. Following a 24 h period, the culture medium was extracted from each well and replaced with fresh, sufficient culture media containing 10% (*v*/*v*) of 10× Presto Blue cell viability reagent (A13262, Invitrogen, Thermo Scientific, Waltham, MA, USA). The cells were incubated for one hour at 37 °C with 5% CO_2_. Variations in cell viability were discovered using fluorescence spectroscopy. The investigations were conducted in triplicate, and the results were presented as a percentage of cell inhibition, with 0% inhibition in the control cells.

#### 3.6.2. Skin Enzyme Inhibition

For this study, the extracts were concentrated by drying after being prepared in 50% ethanol for 6 h with a solid/liquid ratio of 1:20 (*w*/*v*). The powder was then redissolved in 50% (*v*/*v*) ethanol solution, at final concentrations of 5, 2.5, and 1.25 mg mL^−1^. The enzyme inhibition assays were performed for elastase (Fluorimetric kit ab118971, Abcam Plc, Cambridge, UK), collagenase (Colourimetry kit ab139443, Abcam Plc, UK), and tyrosinase (Colorimetric kit ab204715, Abcam Plc, UK). In previous research, the enzyme inhibition experiments were described in detail [51].

### 3.7. Statistical Analysis

Experimental data were fitted using the regression analysis function of TIBCO (Palo Alto, CA, USA) Data Science Workbench (Statistica, Statsoft, Inc., Hamburg, Germany) v 14.0.0.15. The model’s adequacy was determined by assessing the lack of fit, coefficient of determination (R2), and F-test value derived from the analysis of variance.

### 3.8. Economic Viability Study

Energy and reagent costs plus overheads, which accounted for 80% of the costs considered for waste management, equipment depreciation, labor costs, and other expenses, were used to determine the cost of creating XOS-enhanced products. The main assumptions used to calculate the expenses of the manufacturing process are summarized in Table 9 and Table 10. The desired processing rate of 1 kg SCS each batch is the basis for all figures, which are shown in euros. The reagents’ associated costs were calculated (data not shown). Based on its specifications (such as the kind of material, capacity, and power consumption), equipment was picked from industrial suppliers, and data on its power consumption were utilized to estimate energy costs. The expenses for the acquisition of the equipment were not considered in this case study.

## 4. Conclusions

A central composite design was used to investigate the extraction of hemicellulose fractions from sugarcane straws through an autohydrolysis approach. The hemicellulose liquor models revealed that the S/L ratio and temperature had an influence on hemicellulose extraction. Extraction conditions were evaluated in terms of the extraction yield, lack of impurities, and wide molecular weight dispersion. Overall, the optimization of extraction conditions showed that a 1:18 solid/liquid ratio, 165 °C temperature, and 45 min extraction time achieved approximately 24.46% of the yield and a polymerization degree of 17.40.

The XOS-enriched extract exhibited notable antioxidant activity and skin enzyme inhibition. Driven by the phenolic compounds and organic acids present in the extract, these extracts show promising cosmetic potential into anti-aging formulations to protect the skin from oxidative stress and reduce the appearance of wrinkles and age spots. However, due to their cytotoxic effects, they can only be used at low concentrations.

## Figures and Tables

**Table 1:** Characterization of monosaccharide and degradation products in hemicellulose after autohydrolysis and yield of hemicellulose extraction from sugarcane straw using S/L ratio and temperature as variables.

Run #	1	2	3	4	5	6	7	8	9
Ratio S/L	1/8	1/8	1/8	1/8	1/8	1/8	1/8	1/8	1/8
Temp (°C)	150	150	180	180	143	186	165	165	165
Yield liquid fraction (%)	6.07	9.96	22.44	24.34	6.66	21.29	15.54	21.01	16.27
Sugars (g/100 g BM)	1.355	2.372	4.515	3.094	1.534	9.146	0.842	2.695	3.580
Contaminants (g/100 g BM)	0.119	0.237	3.384	3.241	0.175	3.842	0.272	0.274	0.371

**Table 2:** Summary of the effect of temperature (°C) (*X*_1_) and S/L ratio (*X*_2_) on the yield (%) and degradation products according to the factorial experimental design.

Factors	Estimated Effect of	
	Yield	Degradation Products
Intercept	17.553 *	0.395
*X*_1_	12.635 *	2.913 *
*X*_2_	3.293	−0.005
*X* ^2^_1_	−3.482	1.924 *
*X* ^2^_2_	0.399	0.142
*X*_1_ *X*_2_	−0.995	−0.131

* *p* ≤ 0.05.

**Table 3:** Molecular weight analysis of the hemicellulose extractions for time optimization.

Molecular Weight			
Run	MW (kDa)	MP (kDa)	Mn (kDa)
1	120.06	5.81	98.78
2	170.23	75.92	64.41
3	118.17	5.84	17.05
4	119.23	6.56	22.39
5	126.29	5.99	90.62
6	20.96	5.25	5.92
7	119.14	6.18	74.43
8	94.63	6.02	63.00
9	120.64	5.93	66.28
10	94.85	6.14	68.48
11	94.40	5.82	66.54

MW—Molecular weight; MP—Molecular Polydispersity and Mn—number-average molecular weight.

**Table 4:** Characterization of monosaccharide and degradation products in hemicellulose after autohydrolysis and yield of hemicellulose extraction from sugarcane straw using time of extraction as a variable.

Run #	8.1	8.2	8.3	8.4	8.5	8.6
Time (min)	20	20	30	30	60	45
Yield liquid fraction (%)	17.17	17.80	21.98	19.73	24.55	24.46
Sugars (g/100 g BM)	3.315	3.029	3.289	3.605	4.584	3.976
Contaminants (g/100 g BM)	0.241	0.247	0.347	0.274	0.991	0.733

**Table 5:** Composition of hemicellulose extract and molecular weight characterization.

Hemicellulose Characterization				
Composition		Molecular Weight		Yield (%)
Glucose (g/100 g)	12.46 ± 0.25	MW (kDa)	38.44	18.80 ± 0.08
Xylose (g/100 g)	40.33 ± 1.35	MP (kDa)	2.57	
Arabinose (g/100 g)	10.71 ± 0.22	Mn (kDa)	17.49	
Sol. Lignin (g/100 g)	8.35 ± 0.18	DP	116.6	
Acetic Acid	5.34 ± 0.17			

**Table 6:** Composition of the XOS extract and molecular weight characterization.

XOS Characterization				
Composition		Molecular Weight		Yield (%)
Glucose (g/100 g)	9.54 ± 0.05	MW (kDa)	14.11	9.36 ± 0.02
Xylose (g/100 g)	38.46 ± 0.28	MP (kDa)	0.17	
Arabinose (g/100 g)	23.00 ± 0.42	Mn (kDa)	2.58	
Sol. Lignin (g/100 g)	10.60 ± 0.40	DP	17.20	

**Table 7:** Organic acids and phenolic compounds identified in XOS fraction obtained from sugarcane straws.

Compound	TR (min)	Molecular Formula	*m*/*z* Measured [M-H]	MS/MS Fragments (*m*/*z*,)	Hemi. Extract (µg/mg DW Extract)	XOS Extract (µg/mg DW Extract)
Organic acids						
Quinic acid	1.4	C_7_H_11_O_6_	191.00	85	39.84 ± 1.92	63.73 ± 0.06
Malic acid	1.5	C_4_H_6_O_5_	133.01	71, 115	27.17 ± 0.01	48.28 ± 3.35
Dehydroascorbate/Aconitic acid	1.7	C_6_H_5_O_6_	173.01	111	10.97 ± 2.04	14.03 ± 1.31
Azelaic acid	13.3	C_9_H_16_O_4_	187.10	125, 169, 187	10.39 ± 0.53	20.70 ± 2.06
∑					88.37	146.74
Hydroxybenzoic acids						
2,6-Dihydroxyphenylacetic acid	6.2	C_8_H_7_O_4_	167.04	108, 119, 152	1.02 ± 0.19	1.52 ± 0.03
2,3-Dihydroxybenzoic acid	6.6	C_7_H_5_O_4_	153.02	109, 153	0.95 ± 0.04	1.65 ± 0.02
4-Hydroxybenzoic acid	7.9	C_7_H_5_O_3_	137.02	137	1.96 ± 0.12	4.16 ± 0.05
3,4-Dihydroxybenzaldehyde	8.1	C_7_H_5_O_3_	137.02	93, 137	1.77 ± 0.01	4.29 ± 0.02
Vanillaldehyde	8.9	C_8_H_7_O_3_	151.04	108	1.85 ± 0.32	1.68 ± 0.05
4-Hydroxybenzaldehyde	9.3	C_7_H_5_O_2_	121.03	92	18.14 ± 0.03	36.72 ± 0.79
∑					25.69	50.02
Hydroxycinnamic acids						
*p*-Coumaric acid	11.1	C_10_H_9_O_4_	163.04	119	21.45 ± 0.14	31.15 ± 0.26
Ferulic acid	12.0	C_10_H_9_O_4_	193.05	134	2.49 ± 0.00	3.57 ± 0.24
4-O-Feruloylquinic acid	14.5	C_9_H_7_O_3_	367.10	134, 193	0.46 ± 0.02	0.83 ± 0.03
∑					24.41	35.55

**Table 8:** Minimal inhibitory concentration (MIC) and bactericidal (MBC) of the XOS extracts on the inhibition of skin microorganisms.

Microorganism	MIC	MBC
*S. aureus*	-	-
*S. epidermis*	1%	-
*P. aeruginosa*	2%	-
*E. coli*	2%	-
*C. jeikeium*	2%	-
*A. johnsonii*	1%	3%
*C. acnes*	-	-
*C. albicans*	-	-
*T. cutaneum*	1%	-
*R. mucilaginosa*	-	-
*M. furfur*	-	-

MIC—minimum inhibitory concentration; MBC—minimum bactericidal concentration.

**Table 9:** Production costs of hemicellulose liquor. For calculations, electricity costs (EUR/kW.h) of 0.14 were assumed.

Equipment	Power (kW)	Time (h)	Electricity Consumption (kW.h)	Costs (EUR)
Parr reactor	0.5	19.83	9.92	1.40
Chiller Smart H150-2100	2.1	19.83	41.65	5.87
Freeze-dryer	1.78	72	128.16	18.058
Reagents	Quantity (kg)	Price (EUR/kg)		Cost (EUR)
dH_2_O (solvent)	38.993	0.0024		0.0942
Total Costs (EUR/kg Biomass)				25.42
Overall costs	Yield (g hemi/kg straw)	Cost (EUR/g Hemicellulose liquor)		Cost (EUR/kg Hemi)
Hemicellulose liquor	194.66	0.130		130.57

Note: No overheads were considered in the costs.

**Table 10:** Production costs of XOS-enriched extracts. For calculations, electricity costs (EUR/kW.h) of 0.14 were assumed.

Equipment	Power (kW)	Time (h)	Electricity Consumption (kW.h)	Costs (EUR)
Parr reactor	0.5	19.83	9.92	1.40
Chiller Smart H150-2100	2.1	19.83	41.65	5.87
Freeze-dryer	1.78	72	128.16	18.058
Reagents	Quantity (kg)	Price (EUR/kg)		Cost (EUR)
dH_2_O (solvent)	76.493	0.0024		0.1848
Total Costs (EUR/kg Biomass)				25.51
Overall costs	Yield (g /kg straw)	Cost (EUR/g ingredient)		Cost (EUR/kg ingredient)
XOS	93.6	0.273		272.57

Note: No overheads were considered in the costs.

**Table 12:** Variables levels of the temperature and S/L ratio applied in the hydrothermal process optimization.

Run #	S/L Ratio	Temp (°C)	Time (min)
8.1	1/18	165	20
8.2	1/18	165	20
8.3	1/18	165	30
8.4	1/18	165	30
8.5	1/18	165	60
8.6	1/18	165	45

## Data Availability

The data presented in this study are available on request from the corresponding author.

## Supplementary Materials

The following supporting information can be downloaded at https://www.mdpi.com/article/10.3390/molecules30061208/s1, Figure S1. FTIR spectra of XOS extract, different ingredients tested and combination of them: (a) Shea butter, (b) Miglyol 812, (c) Petrolatum, (d) Mineral Oil and (e) Cetiol V.

## References

[1] Pinales-Márquez C.D., Rodríguez-Jasso R.M., Araújo R.G., Loredo-Treviño A., Nabarlatz D., Gullón B., Ruiz H.A. (2021). Circular Bioeconomy and Integrated Biorefinery in the Production of Xylooligosaccharides from Lignocellulosic Biomass: A Review. Ind. Crops Prod..

[2] Lachos-Perez D., Tompsett G.A., Guerra P., Timko M.T., Rostagno M.A., Martínez J., Forster-Carneiro T. (2017). Sugars and Char Formation on Subcritical Water Hydrolysis of Sugarcane Straw. Bioresour. Technol..

[3] Szczerbowski D., Pitarelo A.P., Zandoná Filho A., Ramos L.P. (2014). Sugarcane Biomass for Biorefineries: Comparative Composition of Carbohydrate and Non-Carbohydrate Components of Bagasse and Straw. Carbohydr. Polym..

[4] Brenelli L.B., Bhatia R., Djajadi D.T., Thygesen L.G., Rabelo S.C., Leak D.J., Franco T.T., Gallagher J.A. (2022). Xylo-Oligosaccharides, Fermentable Sugars, and Bioenergy Production from Sugarcane Straw Using Steam Explosion Pretreatment at Pilot-Scale. Bioresour. Technol..

[5] Conab-Página Inicial. https://www.conab.gov.br/.

[6] Aguiar A., Milessi T.S., Mulinari D.R., Lopes M.S., da Costa S.M., Candido R.G. (2021). Sugarcane Straw as a Potential Second Generation Feedstock for Biorefinery and White Biotechnology Applications. Biomass Bioenergy.

[7] Egüés I., Sanchez C., Mondragon I., Labidi J. (2012). Effect of Alkaline and Autohydrolysis Processes on the Purity of Obtained Hemicelluloses from Corn Stalks. Bioresour. Technol..

[8] Scapini T., dos Santos M.S.N., Bonatto C., Wancura J.H.C., Mulinari J., Camargo A.F., Klanovicz N., Zabot G.L., Tres M.V., Fongaro G. (2021). Hydrothermal Pretreatment of Lignocellulosic Biomass for Hemicellulose Recovery. Bioresour. Technol..

[9] Sun D., Lv Z.W., Rao J., Tian R., Sun S.N., Peng F. (2022). Effects of Hydrothermal Pretreatment on the Dissolution and Structural Evolution of Hemicelluloses and Lignin: A Review. Carbohydr. Polym..

[10] Kumar V., Bahuguna A., Ramalingam S., Kim M. (2021). Developing a Sustainable Bioprocess for the Cleaner Production of Xylooligosaccharides: An Approach towards Lignocellulosic Waste Management. J. Clean. Prod..

[11] Valladares-Diestra K.K., Porto de Souza Vandenberghe L., Soccol C.R. (2021). A Biorefinery Approach for Enzymatic Complex Production for the Synthesis of Xylooligosaccharides from Sugarcane Bagasse. Bioresour. Technol..

[12] Kaprelyants L., Zhurlova O., Shpyrko T., Pozhitkova L. (2017). Xylooligosaccharides from Agricultural By-Products: Characterization, Production and Physiological Effects. Food Sci. Technol..

[13] Otieno D.O., Ahring B.K. (2012). The Potential for Oligosaccharide Production from the Hemicellulose Fraction of Biomasses through Pretreatment Processes: Xylooligosaccharides (XOS), Arabinooligosaccharides (AOS), and Mannooligosaccharides (MOS). Carbohydr. Res..

[14] Santos T.M., Alonso M.V., Oliet M., Domínguez J.C., Rigual V., Rodriguez F. (2018). Effect of Autohydrolysis on Pinus Radiata Wood for Hemicellulose Extraction. Carbohydr. Polym..

[15] Garrote G., Falqué E., Domínguez H., Parajó J.C. (2007). Autohydrolysis of Agricultural Residues: Study of Reaction Byproducts. Bioresour. Technol..

[16] Mokhena T.C., Mochane M.J. (2017). Sugarcane Bagasse and Cellulose Polymer Composites.

[17] Costa J.R., Pereira M.J., Pedrosa S.S., Gullón B., de Carvalho N.M., Pintado M.E., Madureira A.R. (2023). Sugarcane Straw as a Source of Arabinoxylans: Optimization and Economic Viability of a Two-Step Alkaline Extraction. Foods.

[18] Melati R.B., Shimizu F.L., Oliveira G., Pagnocca F.C., De Souza W., Anna C.S., Brienzo M. (2019). Key Factors Affecting the Recalcitrance and Conversion Process of Biomass. BioEnergy Res..

[19] Ruiz H.A., Ruzene D.S., Silva D.P., Quintas M.A.C., Vicente A.A., Teixeira J.A. (2011). Evaluation of a Hydrothermal Process for Pretreatment of Wheat Straw-Effect of Particle Size and Process Conditions. J. Chem. Technol. Biotechnol..

[20] Garrote G., Domínguez H., Parajo J.C. (2001). Kinetic Modelling of Corncob Autohydrolysis. Process biochemistry.

[21] Gullón B., Yáñez R., Alonso J.L., Parajó J.C. (2010). Production of Oligosaccharides and Sugars from Rye Straw: A Kinetic Approach. Bioresour. Technol..

[22] Sun X.F., Sun R.C., Fowler P., Baird M.S. (2005). Extraction and Characterization of Original Lignin and Hemicelluloses from Wheat Straw. J. Agric. Food Chem..

[23] Ruiz H.A., Cerqueira M.A., Silva H.D., Rodríguez-Jasso R.M., Vicente A.A., Teixeira J.A. (2013). Biorefinery Valorization of Autohydrolysis Wheat Straw Hemicellulose to Be Applied in a Polymer-Blend Film. Carbohydr. Polym..

[24] Peng F., Ren J.-L., Xu F., Bian J., Peng P., Sun R.-C. (2009). Comparative Study of Hemicelluloses Obtained by Graded Ethanol Precipitation from Sugarcane Bagasse. J. Agric. Food Chem..

[25] Montané D., Nabarlatz D., Martorell A., Torné-Fernández V., Fierro V. (2006). Removal of Lignin and Associated Impurities from Xylo-Oligosaccharides by Activated Carbon Adsorption. Ind. Eng. Chem. Res..

[26] Brienzo M., Carvalho A.F.A., de Figueiredo F.C., Neto P.O., Riley G.L. (2016). Sugarcane Bagasse Hemicellulose Properties, Extraction Technologies and Xylooligosaccharides Production. Food Waste.

[27] Alokika, Anu, Kumar A., Kumar V., Singh B. (2021). Cellulosic and Hemicellulosic Fractions of Sugarcane Bagasse: Potential, Challenges and Future Perspective. Int. J. Biol. Macromol..

[28] Mandelli F., Brenelli L.B., Almeida R.F., Goldbeck R., Wolf L.D., Hoffmam Z.B., Ruller R., Rocha G.J.M., Mercadante A.Z., Squina F.M. (2014). Simultaneous Production of Xylooligosaccharides and Antioxidant Compounds from Sugarcane Bagasse via Enzymatic Hydrolysis. Ind. Crops. Prod..

[29] Kabel M.A., Carvalheiro F., Garrote G., Avgerinos E., Koukios E., Parajó J.C., Gírio F.M., Schols H.A., Voragen A.G.J. (2002). Hydrothermally Treated Xylan Rich By-Products Yield Different Classes of Xylo-Oligosaccharides. Carbohydr. Polym..

[30] de Carvalho D.M., Sevastyanova O., Penna L.S., da Silva B.P., Lindström M.E., Colodette J.L. (2015). Assessment of Chemical Transformations in Eucalyptus, Sugarcane Bagasse and Straw during Hydrothermal, Dilute Acid, and Alkaline Pretreatments. Ind. Crops. Prod..

[31] Kumar N., Goel N. (2019). Phenolic Acids: Natural Versatile Molecules with Promising Therapeutic Applications. Biotechnol. Rep..

[32] Gomez-Molina M., Albaladejo-Marico L., Yepes-Molina L., Nicolas-Espinosa J., Navarro-León E., Garcia-Ibañez P., Carvajal M. (2024). Exploring Phenolic Compounds in Crop By-Products for Cosmetic Efficacy. Int. J. Mol. Sci..

[33] Lima M.D.S., Silani I.D.S.V., Toaldo I.M., Corrêa L.C., Biasoto A.C.T., Pereira G.E., Bordignon-Luiz M.T., Ninow J.L. (2014). Phenolic Compounds, Organic Acids and Antioxidant Activity of Grape Juices Produced from New Brazilian Varieties Planted in the Northeast Region of Brazil. Food Chem..

[34] Panzella L. (2020). Natural Phenolic Compounds for Health, Food and Cosmetic Applications. Antioxidants.

[35] Molina-Cortés A., Sánchez-Motta T., Tobar-Tosse F., Quimbaya M. (2020). Spectrophotometric Estimation of Total Phenolic Content and Antioxidant Capacity of Molasses and Vinasses Generated from the Sugarcane Industry. Waste Biomass Valorization.

[36] Oliveira A.L.S., Carvalho M.J., Oliveira D.L., Costa E., Pintado M., Madureira A.R. (2022). Sugarcane Straw Polyphenols as Potential Food and Nutraceutical Ingredient. Foods.

[37] Kallel F., Driss D., Chaabouni S.E., Ghorbel R. (2015). Biological Activities of Xylooligosaccharides Generated from Garlic Straw Xylan by Purified Xylanase from Bacillus Mojavensis UEB-FK. Appl. Biochem. Biotechnol..

[38] Zhao Y., Chen M., Zhao Z., Yu S. (2015). The Antibiotic Activity and Mechanisms of Sugarcane (*Saccharum officinarum* L.) Bagasse Extract against Food-Borne Pathogens. Food Chem..

[39] Bratcher D.F. (2008). Other Corynebacteria. Principles and Practice of Pediatric Infectious Disease.

[40] Yang X. (2014). Moraxellaceae. Encyclopedia of Food Microbiology.

[41] Prieto-Granada C.N., Lobo A.Z.C., Mihm M.C. (2010). Skin Infections. Diagnostic Pathology of Infectious Disease.

[42] Fournière M., Latire T., Souak D., Feuilloley M.G.J., Bedoux G. (2020). Staphylococcus Epidermidis and Cutibacterium Acnes: Two Major Sentinels of Skin Microbiota and the Influence of Cosmetics. Microorganisms.

[43] Carvalho M.J., Oliveira A.L., Pedrosa S.S., Pintado M., Madureira A.R. (2021). Potential of Sugarcane Extracts as Cosmetic and Skincare Ingredients. Ind. Crops. Prod..

[44] Sluiter A., Hames B., Ruiz R., Scarlata C., Sluiter J., Templeton D., Crocker D. (2008). Determination of Structural Carbohydrates and Lignin in Biomass. Lab. Anal. Proced. LAP.

[45] Dávila I., Gordobil O., Labidi J., Gullón P. (2016). Assessment of Suitability of Vine Shoots for Hemicellulosic Oligosaccharides Production through Aqueous Processing. Bioresour. Technol..

[46] Pablo G., Gullón B., Pérez-Pérez A., Romaní A., Garrote G. (2021). Microwave Hydrothermal Processing of the Invasive Macroalgae Sargassum Muticum within a Green Biorefinery Scheme. Bioresour. Technol..

[47] Gonçalves B., Falco V., Moutinho-Pereira J., Bacelar E., Peixoto F., Correia C. (2009). Effects of Elevated CO_2_ on Grapevine (*Vitis vinifera* L.): Volatile Composition, Phenolic Content, and in Vitro Antioxidant Activity of Red Wine. J. Agric. Food Chem..

[48] Wikler M.A. (2006). Methods for Dilution Antimicrobial Susceptibility Tests for Bacteria That Grow Aerobically: Approved Standard. Clsi Nccls.

[49] Hecht D.W., Citron D.M., Dzink-Fox J., Gregory W.W., Jacobus N.V., Jenkins S.G., Rosenblatt J.E., Schuetz A.N., Wexler H. (2012). M11-A8 Methods for Antimicrobial Susceptibility Testing of Anaerobic Bacteria; Approved Standard-Eighth Edition.

[50] Pfaller M.A., National Committee for Clinical Laboratory Standards (2002). Reference Method for Broth Dilution Antifungal Susceptibility Testing of Yeasts: Approved Standard.

[51] Oliveira A.L.S., Gondim S., Gómez-García R., Ribeiro T., Pintado M. (2021). Olive Leaf Phenolic Extract from Two Portuguese Cultivars –Bioactivities for Potential Food and Cosmetic Application. J. Environ. Chem. Eng..

