# Supplementary material for: Sugarcane Straw Hemicellulose Extraction by Autohydrolysis for Cosmetic Applications
Source: Molecules. 2025 Mar 7;30(6):1208. doi: 10.3390/molecules30061208 (PMC11945048; doi:10.3390/molecules30061208)
Supplement: Supplementary file 1 [file molecules-30-01208-s001.zip › molecules-3485097 - supplementary.pdf]

**Supplementary material:**

Compatibility with cosmetic ingredients

The compatibility of XOS enriched extracts with cosmetic ingredients was evaluated by DSC and FTIR (See figure A1). Physical compatibility of common cosmetic ingredients as representants of major classes of ingredients in cosmetic formulations. Hence, Shea butter was assessed as a thickener agent and emollient; Miglyol 812 (a triglyceride ester of saturated coconut/palm kernel oil derived caprylic and capric fatty acids and plant derived glycerol) as emulsifier; Petrolatum and mineral oil as emollients; and Cetiol V (Decyl Oleate) as a different class of emollient ingredient; finally, phenoxyethanol was characterized as a commonly used preservative ingredient.

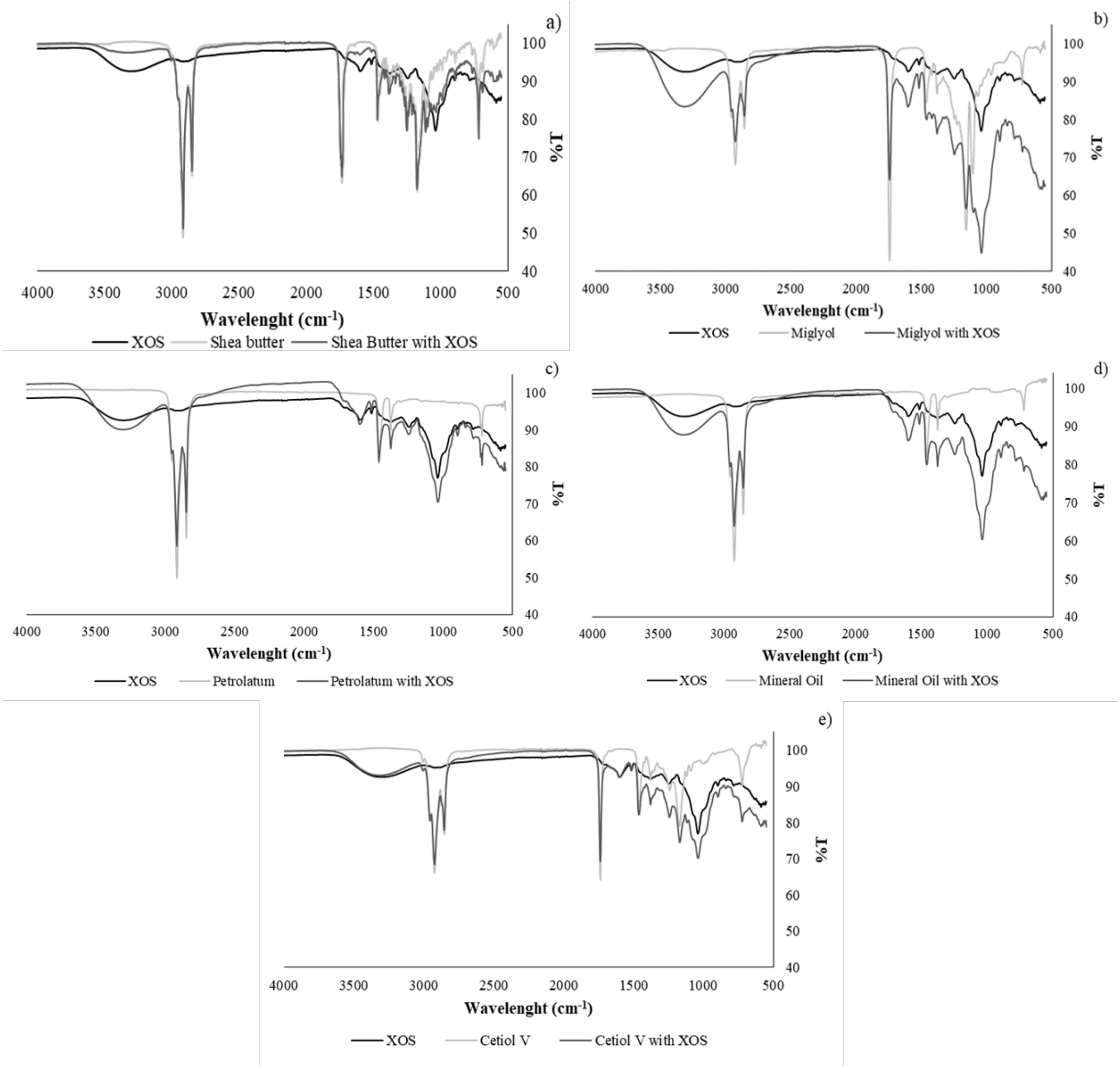


**Figure S1.** FTIR spectra of XOS extract, different ingredients tested and combination of them: a) Shea butter, b) Miglyol 812, c) Petrolatum, d) Mineral Oil and e) Cetiol V.

The characteristic peaks of shea butter (*Vitellaria paradoxa*) that are detectable at 2925 and 2854 peaks attributed to asymmetric and symmetric vibration of $CH_2$ and $CH_3$, in 1724 stretching 1st overtone of -CH2 and 1450 stretching 1st overtone of O-H, 1396 and 1214 stretching of $-CH_2$, 1172 stretching of HC=CH and at 1160 of $-CH_3$. The juxtaposition of peaks of straw peaks of XOS and shea butter shows no chemical interaction between the ingredients.

Miglyol 812 (a triglyceride ester of saturated coconut/palm kernel oil derived caprylic and capric fatty acids and plant derived glycerol) has characteristic IR bands at 1022-1260 $cm^{-1}$ attributed to C-O-C stretching, 1292 $cm^{-1}$ (C-CO-O) and 2870-2960 $cm^{-1}$ corresponding to $CH_3$ stretching. Also, the characteristic peak of XOSX from straw at 1725 $cm^{-1}$ that represents the presence of acetyl group

(C=O) of hemicellulose is also detectable in both isolated and mixed spectra. Therefore, it is not datable any incompatibility between these ingredients.

The large absorption band of petrolatum between 3000 and 2800 $cm^{-1}$ likely corresponds to the C–H stretching vibrations of the $–CH_3$ and $–CH_2–$ groups. The peaks at 1465 and 1382 $cm^{-1}$ are attributed to the asymmetrical and symmetrical vibrations of the $–CH_3$ group, respectively. A comparison of the absorption peaks indicates a close association between petrolatum and the XOS compounds derived from straw, suggesting potential incompatibility between these ingredients.

Mineral characteristic IR bands at 1373 and 1465 $cm^{-1}$ attributed to $CH_2$ and $CH_3$ stretching, and 2925 and 2854 peaks attributed to asymmetric and symmetric vibration of $CH_2$ and $CH_3$ and at 3008 -CH vibration of olefinic hydrogen. There is detectable an incompatibility between mineral oil and XOS from straw due to arise of a higher intensity peak at 1727 cm-1 due represents the presence of acetyl group (C=O) of hemicellulose.

Cetiol V (decyl oleate) characteristic signal at 725 $cm^{-1}$ ($CH_2$ rocking), 1097 $cm^{-1}$ (O-CH2-C), and 1292 $cm^{-1}$ (C-CO-O); and 2851and 2918 $cm^{-1}$($CH_2$ sym. And a sym. stretch) was in both spectra of isolated and mixed ingredients. Also, the characteristic peak of XOS from straw at 1725 $cm^{-1}$ that represents the presence of acetyl group (C=O) of hemicellulose is also detectable in both isolated and mixed spectra. Therefore, it is not datable any incompatibility between these ingredients.

The characteristic peaks of phenoxyethanol are detectable in FTIR, at 3030-3090 the $-CH_3$ and $-CH_2$ stretch, at 1497 and 1599 the C=O aromatic linkage, and the COC ether bond at 1082 and 1246 are detectable in the isolated phenoxyethanol spectra and upon mixing with XOS straw. Also, the XOS peaks (at 1725 $cm^{-1}$ and 1516 $cm^{-1}$) from straw are able to be distinguished in the mixed spectra however in lower intensity due to concentration issues. Therefore, it is not detectable any physico-chemical incompatibility between XOS ingredients and phenoxyethanol as a preservative ingredient common in cosmetic formulations.
